# Understanding barriers and facilitators to implementation of a patient safety bundle for pregnancy-related severe hypertension in 3 North Carolina outpatient clinics: a qualitative study

**DOI:** 10.1186/s43058-024-00685-7

**Published:** 2025-01-09

**Authors:** Aparna G. Kachoria, Hiba Fatima, Alexandra F. Lightfoot, Linda Tawfik, Joan Healy, Asia Carter, Narges Farahi, E. Nicole Teal, Joumana K. Haidar, Herbert B. Peterson, M. Kathryn Menard

**Affiliations:** 1 Department of Maternal and Child Health, University of North Carolina Gillings School of Global Public Health, Chapel Hill, NC 27599 USA; 2 Department of Health Behavior, University of North Carolina Gillings School of Global Public Health, Chapel Hill, NC 27599 USA; 3 Department of Family Medicine, University of North Carolina School of Medicine, Chapel Hill, NC 27599 USA; 4 Department of Obstetrics and Gynecology, Division of Maternal and Fetal Medicine, University of North Carolina School of Medicine, Chapel Hill, NC 27599 USA

**Keywords:** Implementation science, Preeclampsia, Respectful care, Rural maternal health, United States, Intervention, Barriers, Facilitators

## Abstract

**Background:**

Pregnancy related hypertension is a leading cause of preventable maternal morbidity and mortality in the US, with consistently higher rates affecting racial minorities. Many complications are preventable with timely treatment, in alignment with the Alliance for Innovation on Maternal Health’s Patient Safety Bundle (“Bundle”). The Bundle has been implemented successfully in inpatient settings, but 30% of preeclampsia-related morbidity occurs in outpatient settings in North Carolina. To address this, we have integrated community engagement and implementation science approaches to identify facilitators and barriers to Bundle implementation, which supports its adaptation for outpatient settings and identifies implementation strategies to be tested in a subsequent study.

**Methods:**

Eleven key informant interviews were conducted across three clinics to assess the implementation needs for effectively utilizing the Bundle. The interview guide was created using the Consolidated Framework for Implementation Research domains to identify facilitators and barriers to implementation. Additionally, three focus group discussions with patient participants were conducted to understand lived experiences and perceptions of respectful care. A coalition of community partners, patients, providers, those with lived experience, and the research team reviewed materials from the formative study design to dissemination and planning for future study.

**Results:**

Barriers included inadequate provider-patient interaction time, patients’ lack of transportation to access care, limited protocols to inform/assess/treat/escalate patients, and workforce capacity (staff training and turnover). Facilitators included staff recognition of the importance of treating preeclampsia, champion buy-in of the Bundle’s ability to improve outcomes, co-location of pharmacies for immediate treatment, and staff capacity. Respectful care principles were repeatedly identified as a facilitator for Bundle implementation, specifically for patient awareness of preeclampsia complications and treatment adherence.

**Conclusions:**

Findings highlight the importance of community-engaged approaches. Further, clinic staff regarded Bundle implementation as crucial for the outpatient setting. Identified barriers suggest that strategies should address systemic social supports (i.e., transportation, childcare) and improve access to and use of home blood pressure monitoring. Identified facilitators support improving communication, increasing clinic champion engagement, enabling systems for identifying at-risk patients, and training staff on accurate blood pressure measurement. Successful Bundle implementation requires addressing systemic barriers to delivering respectful care, such as limited time with patients.

Contributions to the Literature

- Implementation frameworks that assess contextual factors that can impact maternal health interventions like the Bundle are vital to effective implementation in outpatient settings.
- Respectful care was identified as a facilitator for recognizing and treating severe hypertension in pregnancy.
- Barriers to Bundle implementation include systemic issues such as limited clinician-patient interaction and patient childcare and transportation needs that limit patient access to care.
- Facilitators of Bundle implementation include co-located pharmacies with needed treatment medications and clinic champions who buy into the Bundle's importance.

## Background

Despite significant innovation in maternal and child health (MCH), maternal morbidity and mortality rates are higher in the United States (US) than in any other high-income country, with substantially higher rates among some racial minorities, such as Native American and non-Hispanic Black women compared to white women [1]. An important contributor to maternal morbidity and mortality is hypertension in pregnant and postpartum women, which has increased by 25% in the last thirty years [2].

A review of data from Maternal Mortality Review Committees (MMRCs) in 36 states found that hypertensive disorders of pregnancy contribute to 6.5% of all maternal deaths [3]. Preeclampsia occurs in about one in 25 pregnancies in the US, and Black women suffer a 60% higher rate of in-hospital hypertension-related mortality [4–6].

Health outcomes pertaining to pregnancy-related hypertension vary between racial and ethnic groups, raising critical equity concerns for MCH programs and policies [3]. One study of 56,000 births in North Carolina (NC) found that 13% of women experienced hypertension during their pregnancy [7]. The NC Maternal Mortality Review Committee also lists hypertension during pregnancy as a cause of preventable deaths and recommends more education for providers on this issue [8]. Lastly, persisting inequalities in clinical outcomes related to hypertension during pregnancy call for equity-informed implementation strategies.

The Alliance for Innovation on Maternal Health (AIM) released the Severe Hypertension During Pregnancy and Postpartum Patient Safety Bundle (Bundle)—an evidence-based intervention to synergize and standardize care for women with hypertension during and after pregnancy [9]. The Bundle comprises five components: Readiness, Recognition, Response, Reporting, and Respectful Care (5 Rs). Importantly, AIM made the decision to add Respectful Care to the Bundle during our study, further highlighting the importance of examining respectful care and ensuring it is included in maternal health safety interventions to advance equity “for all patients in every setting from every provider” [9].

There is evidence that this intervention is effective in preventing adverse outcomes for pregnant people with hypertension in the inpatient setting [10]. However, the impact of the Bundle has yet to be determined in the outpatient setting. This study aims to support this determination by utilizing principles, frameworks, and tools from implementation science to not only identify facilitators and barriers to implementing the Bundle in outpatient settings but also to support its successful adaptation to these settings for the improvement of maternal health outcomes in the United States [11, 12].

Additionally, community-based participatory and community-centered approaches are increasingly important contributors to maternal and child health research. Community-engaged approaches also decrease gaps in health equity and should be considered in approaches to reducing morbidity and mortality [13]. A North Carolina Medicaid study found that community involvement significantly increased positive outcomes in maternal health [14]. Documenting the role of community involvement in implementing the Bundle will offer valuable insights and innovative solutions to current problems in outpatient obstetric care.

This study sets the foundation for a larger trial of implementation strategies to adapt, implement, and sustain the Bundle in the outpatient setting. This study aims to better understand how implementing interventions for improving maternal health outcomes can be anchored in community-centered approaches and how the identified facilitators and barriers can drive future implementation in similar outpatient settings.

## Methods

This research is formative work wherein the Bundle was adapted for use in the outpatient setting to recognize and respond to severe hypertension in pregnancy. The concept was a component of quality improvement initiatives of the North Carolina Perinatal Region IV Clinical Champions. Community-based providers in the region called for support to improve severe hypertension outcomes in their settings. Consistent with the implementation science literature, we sought to identify and better understand the factors that could facilitate or hinder Bundle implementation in the outpatient setting by using the Consolidated Framework for Implementation Research (CFIR) to guide our inquiry and assess contextual factors potentially affecting implementation [11, 15]. Findings were intended to inform a larger follow-up trial of implementation strategies (a hybrid type 3 implementation trial). In response to ongoing quality improvement initiatives of Perinatal Region IV Champions,). Three facilities with an established trust relationship with the study team were chosen to participate. These federally qualified health centers (FQHCs) are in rural counties in central North Carolina, and all are part of one multicenter FQHC health system. The diverse patient population living in rural areas experiences higher rates of hypertensive disorders of pregnancy and are at higher risk for pregnancy-related morbidity and mortality, which we aimed to address.

Additionally, this health system is an essential community partner, working to serve patients in hard-to-reach areas throughout the counties where the clinics are located. A representative from this health system was involved in the study coalition to drive community engagement and met regularly with one of the study’s Principal Investigators for alignment and understanding. The health system assisted in identifying and establishing provider champions in the three clinics, and these champions met routinely to discuss the study and guide the implementation.

After receiving IRB approval, two sources of qualitative inquiry were used to explore determinants of implementation, with a focus on three components of the Bundle—Readiness, Recognition, and Response. We used Key Informant Interviews (KIIs) to gain insight from clinic providers and staff and conducted Focus Group Discussions (FGDs) to understand the perspective of patients. The guides for KIIs and FGDs were developed concurrently. Guides were developed jointly by the research team and the clinic champions to encourage all participants to share perspectives about the Bundle in their clinic's context. The use of CFIR domains to structure the KII guide and formulate its questions is summarized in Table 1**.** Guides were also developed with respectful care in mind with the goal of listening to how patients and providers perceive respectful care.

**Table 1:** CFIR domains and corresponding KII interview guide questions

CFIR Domain	KII Question Example
**Intervention characteristics:** the intervention source, its adaptability, and the quality and validity of evidence that the intervention will have desired outcomes	What is your view of the advantages of implementing this bundle versus the current practice?
**Inner setting:** structural characteristics, clinic culture, implementation climate, such as compatibility and relative priority of the intervention, and readiness for implementation	Tell me about current clinic protocols for the diagnosis of hypertension during pregnancy and the post-partum period?
**Outer setting:** extent to which the organization understands and prioritizes patient needs and barriers and facilitators to meet those needs	Of your patients with severe hypertension, can you please suggest any factors that could affect, in a positive or negative way, the proper management of the condition at the outpatient clinic?
**Individual characteristics:** knowledge and beliefs about the intervention, self-efficacy, and their individual stage of change in adopting the intervention	To what extent do you believe in your own capabilities to implement actions needed to successfully adopt the bundle?
**Process:** perceptions and ideas related to planning, engaging appropriate individuals, executing, and evaluating the innovation implementation	What would be ideal ways to scale up the successful implementation of the bundle?

Specifically, the KII guide included questions about readiness to implement this Bundle in the outpatient setting and visual aids to help the interviewee understand the Bundle and consider how it would fit into their clinic's processes and context. Beyond the CFIR domains, the KIIs included questions to better understand provider and staff perspectives on respectful care in general and as they relate to the Bundle implementation.

KII participants were selected from the pilot clinics through a nomination process; clinic champions nominated four potential staff for voluntary KII participation (60–90 min) that fit the selection criteria (management level, clinical level, administrative level), with the final distribution as follows:

- One Director/Manager
- Two clinical care team members (a physician, certified nurse midwife, advanced practice provider, nurse, or medical assistant)
- One administrative support staff (care coordinator, pharmacy technician, or front desk staff)

Thirteen potential KII participants were nominated, from which 11 KIIs were conducted across the three clinics. Informed consent was sought at the start of each interview. Interviews were conducted virtually and recorded by a team of two research staff. After transcripts were generated from recordings and any identifying information removed, the recordings were deleted. Four members of the research team analyzed the KII data using the RaDAR technique for rapid qualitative analysis [16]. The research team met bimonthly to review and ensure reliability across analyses.

FGDs were conducted with patients to better understand their experiences, their understanding of the proposed HTN Bundle, and their perspective on respectful care. The FGD guide was constructed using the CFIR framework, with a focus on the Outer Setting domain, to best understand patient needs and resources and identify a participant-driven shared definition of respectful care. Questions were designed to identify patient knowledge and attitudes towards hypertension in pregnancy and to understand patient-defined respectful care and barriers and facilitators to care. A visual representation of the care process was also shared with participants to help patients recall their own care experiences. Patients were also asked about preferred methods and timing of receiving information on hypertension during pregnancy.

FGDs were conducted by a member of the study team together with a trained focus group facilitator who was racially/ethnically/linguistically concordant with the patient participants. Patients for the focus groups were from the counties in the catchment areas of the pilot clinics or patients treated for severe hypertension in the referral facility serving the catchment area. Eligible participants included female patients either living near or receiving care in the three pilot clinics between 18–44 years of age who were at least 28 weeks pregnant at the time of data collection (October 2021). Electronic health record data were used to identify eligible participants who were sent recruitment letters and a link for consent and completion of demographic information. Additionally, focus group members were required to fill out an initial online informed consent form, which may have been an additional barrier.

After enrolling online, patients received scheduling calls and participated in the FGDs per their race/ethnicity/language preferences. Some patients opted to be notified via telephone or email, the latter of which had the lowest response rate. The FGD composition was as follows:

- One focus group with women who were treated for severe hypertension at the facility that serves the catchment area for the FQHCs (*n* = 4)
- One focus group with self-identifying Black/African American patients who received care at the participating FQHCs (*n* = 2)
- One focus group scheduled with Spanish-speaking patients receiving care at the FQHCs. Due to no-shows or other commitments, the total number of participants in this group was one, so the FGD was modified into a discussion session to still gather the Spanish-speaking patient’s perspective

FGDs were recorded using Zoom after informed consent was obtained. Participants were anonymized on Zoom to protect their identities during the session, and analysts were unaware of participant names when analyzing the data. To improve transcription quality, transcripts of the Zoom recordings were obtained and submitted through Otter.ai. The recordings were deleted after the research team reviewed the transcripts and redacted identifying information. The research team utilized the same RADaR approach for organizing and synthesizing FGD participant responses [16].

Similarly, data collection through the KIIs and FGDs occurred concurrently without one method influencing the other. The goal was to employ both methods to best capture facilitators and barriers, while anchoring a community-engaged approach, and prioritizing respectful care.

## Results

### Study participant characteristics

#### Clinic demographics

The three clinics are FQHCs in two predominantly rural counties in Central North Carolina. The clinics serve a diverse patient population, predominantly low-income rural residents, with high Black/African Americans and Latinx representation. Across this multicenter FQHC network, nearly 88% have incomes at 100% or less of the federal poverty limit; nearly 50% are Hispanic/Latino and 24% Black/African American; 55% are uninsured and 23% have Medicaid. Almost 40% are best served in a language other than English [17].

#### KII and FGD participant demographics

The 11 KII participants were all providers or staff in one of the three clinics. The seven FGD participants across three groups were members of the community, and most received prenatal care at one of the three clinics (there were five participants receiving prenatal and/or postpartum care outside the study health system). The focus group members spanned multiple ages (20–41 years old), race and ethnicities (White, Black, and Hispanic), insurance status, income level, and education. KII participant characteristics are in Table 2. Due to the low sample size of the focus groups, participant characteristics are shared in aggregate to protect participant privacy.

**Table 2:** KII participant characteristics

KII Participant Characteristics (*n* = 11)	
Variable	**Total** ***N***
Clinic Breakdown	
Clinic 1	4
Clinic 2	4
Clinic 3	3
Interviewee Role^1^	
Director/Manager	3
Clinician	8
Pharmacy Technician	1
Years in Role	
6 months – 1 year	5
2–5 years	4
5–10 years	1
> 10 years	1
Gender^2^	
Female	10
Male	1
Age	
25–34 years	3
35–44 years	6
45–54 years	1
> 55 years	1
Education	
High School	3
Associate Degree	1
Professional Degree or PhD	4
Master's Degree	3
Race	
White	7
Black/African American	2
Other	2
Ethnicity	
Hispanic	3
Not Hispanic	8

^1^Some interviewees have multiple roles within the clinic, so the total exceeds 11 for this category

^2^Gender preferences included man, woman, transgender, gender nonconforming/non-binary, and a prefer to self-describe

### Determinants to implementation

The results indicate clear facilitators and barriers to implementation as outlined and defined using the CFIR framework. Specifically, these results identified facilitators and barriers to recognizing and responding to severe hypertension and addressed the study objectives of understanding these factors in the context of community-engaged care. The KII results emphasized a need for a standardized protocol for identifying and managing patients with severe hypertension during pregnancy. Other findings provided details on how to best accomplish this.

Respondents highlighted that respectful care, defined by providers as listening to patients and being compassionate, is crucial to intervention implementation. Barriers to respectful care and timely treatment include limited time for clinician-patient interaction and patients’ lack of transportation to clinics. Systemic barriers include the inability to schedule timely appointments to assess warning signs and the lack of an algorithm to determine when to escalate care. Facilitators included co-location of pharmacies (i.e., when the pharmacy is located within the clinic) for medications when severe hypertension was identified and clinic champion buy-in of the importance of Bundle implementation. Other barriers stemmed from workforce capacity issues related to staff training, turnover, and workload. Results are further presented based on clinic-specific responses and matched to CFIR domains below in Tables 3 and 4.

**Table 3:** Clinic-specific Barriers and Associated CFIR Domains

Barriers by Clinic and Associated CFIR Domain(s)		
**Site 1**	**Site 2**	**Site 3**
Staffing issues & staff burnout and cynicism which has led to large staff turnover	Delayed treatment: There is a delay between the medical assistant measuring BP and provider becoming aware of patient’s high BP	Patient constraints such as lack of childcare and non-traditional work hours challenge the clinic with follow-up appointments
CFIR Domain: Inner Setting	CFIR Domain: Inner Setting	CFIR Domain: Outer Setting
Few staff to manage prenatal care	Difficulty in patients being able to get an appointment quickly enough due to getting through by phone to the clinic; or patients experience long wait times on the phone and are unable to connect directly to the triage nurse	Patient’s access to care – and general awareness of what to do in a severe HTN scenario
CFIR Domain: Inner Setting	CFIR Domain: Outer Setting, Inner Setting	CFIR Domain: Outer Setting
Patient’s previous experience dictating their ability and desire to follow provider instructions	Inaccurate BP measurements due to time constraints of clinic workflow	Provider instructions are not always understood by the patient
CFIR Domain: Outer Setting	CFIR Domain: Inner Setting	CFIR Domains: Inner and Outer Settings; Process

**Table 4:** Clinic-specific Facilitators and Associated CFIR Domains

Facilitators by Clinic and Associated CFIR Domain(s)		
**Site 1**	**Site 2**	**Site 3**
Protocols for how clinic staff can communicate and follow up with patients postpartum	Onsite clinic offering cheaper hypertension medications so patients can get them during their visit (colocation of medications)	Offering patients appointments after hours
CFIR Domain: Inner Setting, Process	CFIR Domain: Inner Setting	CFIR Domain: Inner and Outer Setting
Pharmacy team on site	Some existing clinic protocols already facilitate increased communication between Medical Assistant and provider to ensure quicker measurements for BP	Provider care team is a combination of residents (physicians in training) and attendings (fully trained physicians), which has led to good care
CFIR Domain: Inner Setting	CFIR Domain: Inner Setting	CFIR Domain: Inner Setting

### Respectful care

Though respectful care was not initially part of the AIM Bundle at the time of the initiation of this study, we included it in the FGD guides, where it was a recurring theme. Respectful care also organically arose in responses from both KII and FGD participants. The KIIs were rooted in understanding the clinic’s perspective of improving implementation to improve maternal health outcomes, with respectful care as a facilitator. Providers shared their definitions and perspectives during KIIs and during patient FGDs participants described “What is Respectful Care” and “What Isn’t Respectful Care.” Respectful care was also emphasized as an essential facilitator for implementing the Bundle. Most notably, all FGD respondents agreed that being dismissed or treated condescendingly was the antithesis of respectful care. Both KII and FGD participants emphasized the need for effective communication in understanding how to manage and treat potential hypertension:

“I think most of [effective hypertension management] has to do with respectful compassionate communication with the patient. I literally just had this scenario last week where mom came in with a blood pressure of 168 systolic and a repeat of 160. And she had the previous week had mild range blood pressure. So, really communicating with her and her husband, that she has severe range blood pressures and that she needed a higher level of care was really helpful…sitting down with her and letting her know why we were concerned, and what the plan of care was, and that the goal was to keep her and her baby safe and healthy, that seemed to really speak volumes to her.” *- Provider*

Communication was again emphasized through the discussion of respondents and discussed by all informants. All spoke about communication generally and specifically, the idea that people felt safe, comfortable, and not alone. Communication was highlighted unanimously by all informants. One interviewee shared another perspective regarding staff biases:

“The clinic, in general, has a very familial feel …so people are very familiar with the staff, usually, and the providers… there's an assumption of respect, but I think that there is… a danger in our clinics having a little bit of Saviorism where we're like, “Look at us, doing this care for these needy patients. I haven't seen a standardized way in which we ensure… [respectful] care….I think a big [way to provide respectful care] would be the ability for patients to contact a knowledgeable person in a timely way about their pregnancy or postpartum period. It doesn't have to be a provider but having a triage nurse who is specific for that population who they can easily access would mean the world*. - Provider*

Another noted the connection between respectful care as a patient right embedded within human rights as a whole:

“The term respectful care, especially in the context of maternity care is really important and…[comes] out from the Human Rights Framework – to be free from harm in treatment…developing trauma-informed frameworks [are] really helpful here. Respecting someone’s choices and preferences, especially if they don’t align with your recommendations.” *–Provider*

Regarding implementation specifically, one respondent shared ideas to facilitate respectful care within the clinic to ensure care needs are met in a respectful manner:

“…I almost think the reporting and the respectful care should be like weaved in, I think a case-based sort of approach would definitely help adopting elements serially instead of justone at a time. Respectful care in helping patients recognize or staff recognize respectful care and how to respond…how can we focus on respectful care while responding, or how can we ensure that we are ready in a way that means something to us systematically.” -*Provider.*

The focus groups created space for patients and those in the community with lived experience to provide strong insight into what respectful care means to them:

*“Whenever I have a concern or a question? The doctor actually listens and answers and* *not just says, ‘Oh, you're fine.’ So, being listened to and not ignored to my concerns is what* *I feel [is respectful care].”—Patient.*

*“The nurses were there. I was never alone for a moment.” - Patient*

*“Most of my care I received through a midwife, and I will say all my interactions there you* *know I was part of the conversation I was always asked what my personal goals were,* *what my concerns were what I wanted out of my pregnancy, that kind of thing. And in* *that instance, in that setting, I always felt very comfortable.”—Patient.*

In addition to explicitly defining respectful care, some of the participants also emphasized what respectful care is not:

*“I think I said to my husband after one appointment that the midwife treated me like I was the stupidest kindergartener in the class...she wanted me to ask questions, but when I did, she made me feel like I was dumb...So, I didn't appreciate that. I don't like big being called Mommy, I have a name.” - Patient*

**Fig. 1:**
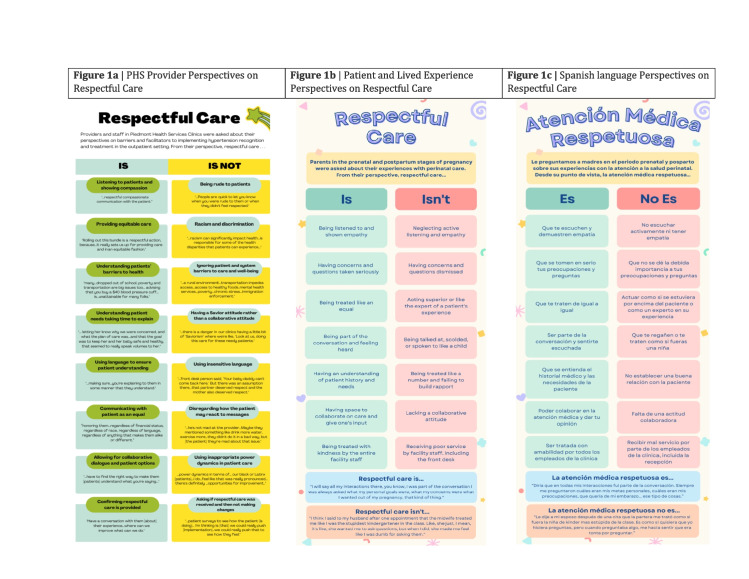
**a** PHS Provider Perspectives on Respectful Care. **b** Patient and Lived Experience Perspectives on Respectful Care. **c** Spanish language Perspectives on Respectful Care

A panel of infographics summarizes what respectful care means from the provider perspective (Fig. 1a), the patient perspectives (Fig. 1b), and the Spanish language version (Fig. 1c).

An overarching theme from the KIIs and the FGDs was the importance of community engagement throughout the study. From data instrument creation to data collection, patient perspectives and community voices were integral to understanding the facilitators and barriers to Bundle implementation. Additionally, community engagement was a vital component in defining respectful care and understanding what respectful care means and looks like from different perspectives.

Key strategies including access to improved care at home (i.e., blood pressure cuffs) and clinics (i.e., tailored training for providers) were identified for future implementation efforts, summarized in Table 5.

**Table 5:** Key facilitators for successful implementation

Facilitators for health systems and patients
**Clinic specific strategies**
Identify and engage clinic champions
Enhance and support a multidisciplinary team with multiple services—nurse triage, co-located pharmacies, dieticians, and ancillary services (i.e., social workers and behavioral health specialists)
Improve front desk staff communication and roles relative to Bundle implementation and clinic philosophy
Tailor training, including simulations, for clinic staff, in advance and during implementation
Ensure proper documentation of patient history, especially for those with hypertension
**Patient specific strategies**
Provide patients with improved access to blood pressure monitoring, such as home use blood pressure cuffs
Distribute standardized educational materials, available in English, Spanish, and any other language that would be helpful for patients
Address social determinants of health, such as transportation, childcare, and housing, and the specific nuance with which these affect patients, individually
Determine what kind of social support patients have (i.e., familial support, support from friends etc.)

These strategies emphasize solutions that work across all domains of the CFIR, but specifically the Inner Setting, Outer Setting, and Process domains.

## Discussion

This study, to our knowledge, is the first of its kind for utilizing community-engaged perspectives to identify barriers and facilitators for implementing the Bundle to inform its adaptation in the outpatient setting. Additionally, utilizing community engagement methods and building partnerships in this study is helpful for setting a strong foundation for future Bundle implementation efforts. Using a community coalition to ensure materials were designed and later disseminated effectively, for example, was one facet of this study that could be used to inform future studies—not just within the field of pregnancy and postpartum hypertension, but in maternal and child healthcare in general.

Care coordination and implementation of successful care practices for improved care delivery continue to be important both within and beyond prenatal and postpartum care. Myriad factors positively and negatively affect a clinic’s ability to implement the Bundle and thereby improve hypertension-related mortality and morbidity in pregnancy. Understanding these factors in the context of CFIR provides an improved understanding of the facilitators and barriers to intervention implementation, with implications for outpatient care.

Importantly, these results and the facilitators and barriers identified in both the interviews and focus group discussions support other research about what respectful care is and why it is an important facilitator for success in Bundle implementation [18, 19]. As noted in their Cycle to Respectful Care, Green et al. share a tool that can be used to help providers implement respectful care practices within their clinics [18]. Jolivet et al. have studied the operationalization of the respectful maternity care framework at the provider level, finding that respectful care is a facilitator but difficult to implement given structural barriers and lack of operational definitions [19].

The implementation strategies identified corroborate findings in other areas of public health, especially related to the systemic barriers surrounding access to care [20, 21]. For example, improving access to care through clinic and patient interventions such as improving provision of patient education materials and use of home blood pressure monitoring can help alleviate this burden. Our results further highlight the need to keep respectful care at the forefront of implementation activities. Implementation across settings is a large endeavor, and findings from this study offer promising results about acceptability and feasibility moving forward. Building upon existing clinic cultures can improve care coordination and delivery, and incorporating elements of respectful care intentionally will not go unnoticed by patients.

### Strengths

This study’s strengths include its novelty in incorporating both community-engagement and implementation science approaches to assess barriers and facilitators to reducing morbidity from pregnancy-related hypertension in the outpatient setting. Specifically, we used implementation frameworks (CFIR) to develop the KIIs guide and determine domains that need improvement. Further, we used community-engagement to gain perspectives from rural health providers and patients in understanding barriers to access to care. Another strength of this study is the use of implementation science concepts to ensure and document the adaptation and implementation of the Bundle in three rural outpatient clinics, which sets the foundation for future testing and expansion in the upcoming larger clinical trial of implementation strategies [22]. Further details about the formative work of Phase I are discussed in forthcoming research (Lightfoot AF, Farahi, N, et al: Community-engaged, Equity and Implementation Science-Informed Methods to Adapt a Severe Hypertension in Pregnancy Safety Bundle and Prepare for Implementation in the Outpatient Setting, under review).

### Limitations

While this study has many strengths, there are some limitations. Namely, both KII and focus group sample sizes were small. It is important to note that recruiting focus group participants was particularly challenging due to logistical issues during the COVID-19 pandemic. However, the insights into facilitators and barriers from the clinic and patient perspective provided important contextual information about considerations for Bundle implementation in outpatient settings. While not broadly generalizable, what we learned from these qualitative findings provided perspectives from clinic providers and staff and birthing people on the centrality of respectful care as crucial to improving care in the outpatient settings in which they work. Additionally, defining respectful care is an evolving process and may be interpreted in different settings by different groups. However, these limitations highlight the need for a community-engaged approach to determining what respectful care is, and adhering to respectful care principles in the delivery of interventions.

Another limitation of the study was that data collection took place during the COVID-19 pandemic, which severely impacted the availability of respondents. However, the data we collected from those able to participate provided valuable insight into respectful care and how to implement it more effectively in the clinical setting.

Centering respectful care is an important way to improve health equity, especially for pregnant people experiencing hypertension and seeking treatment in the outpatient setting.

## Conclusions

Community perspective was an integral part of this study, and the understanding gained from working with those with lived experience, patients, and providers, was beneficial in informing implementation strategies for future studies that will expand on this formative work. Barriers, including workforce capacity, delays in recognition of and response to severe hypertension, and patient constraints related to systemic problems around access to care, transportation, and childcare should be addressed to improve prenatal care. Facilitators that should be emphasized and implemented include standardizing clinical care protocols, improving access to treatment through co-located pharmacies, and expanding access to care by increasing available hours for clinic appointments. Notably, in using a community-engaged approach, respectful care arose as an integral topic for both patients and providers, lending further support to the facilitators identified in this study. Based on these results, we intend to study the identified barriers by further refining implementation strategies that intensify focus on respectful care, including expanding our coalition, implementing patient action groups, providing more clinical staff training and coaching (including simulation activities), and relevant, accessible patient education materials. Findings from this study will enable further research of respectful care and implementation strategies such as training, coaching, and simulation. The next phase of our work will test these implementation strategies in 20 clinics located in four central North Carolina counties to ultimately adapt for widescale dissemination with the goal of improved maternal health outcomes via reduced morbidity and mortality from severe hypertension in pregnancy [22].

## Data Availability

Data are available upon reasonable request.

## Abbreviations

CFIR: Consolidated Framework for Implementation Research
FGD: Focus Group Discussion
FQHC: Federally Qualified Health Center
HTN: Hypertension
KII: Key Informant Interview
MCH: Maternal and Child Health
MMRC: Maternal Mortality Review Committee
PHS: Piedmont Health Services
